# Gender differences in fatigability and muscle activity responses to a short-cycle repetitive task

**DOI:** 10.1007/s00421-016-3487-7

**Published:** 2016-10-14

**Authors:** Divya Srinivasan, Kathryn E. Sinden, Svend Erik Mathiassen, Julie N. Côté

**Affiliations:** 1 Department of Occupational and Public Health Sciences, Centre for Musculoskeletal Research, University of Gavle, 801 76 Gavle, Sweden; 2 McGill University, 475 Pine Avenue West, Montreal, QC H2W 1S4 Canada; 3 CRIR Research Centre, Jewish Rehabilitation Hospital, 3205 Alton Goldbloom Place, Laval, QC H7V-1R2 Canada; 4 Grado Department of Industrial and Systems Engineering, Virginia Polytechnic Institute and State University, Blacksburg, VA 24060 USA

**Keywords:** Pointing movement, Motor variability, Multi-jointed movements, Shoulder–elbow coordination, Motor control, Cycle-to-cycle variability

## Abstract

**Purpose:**

Epidemiological research has identified women to be more susceptible to developing neck–shoulder musculoskeletal disorders when performing low-force, repetitive work tasks. Whether this is attributable to gender differences in fatigability and motor control is currently unclear. This study investigated the extent to which women differ from men in fatigability and motor control while performing a short-cycle repetitive task.

**Methods:**

113 healthy young adults (58 women, 55 men) performed a standardized repetitive pointing task. The task was terminated when the subject’s perceived exertion reached 8 on the Borg scale. The time to task termination, and changes in means and cycle-to-cycle variabilities of surface electromyography signals from start to end of the task, were compared between women and men, for the upper trapezius, anterior deltoid, biceps and triceps muscles.

**Results:**

Women and men terminated the task after 6.5 (SD 3.75) and 7 (SD 4) min on average (*p* > 0.05). All four muscles showed an increase of 25–35 % in average muscle activity with fatigue (no significant sex differences). However, men exhibited a higher increase than women in trapezius muscle variability with fatigue (31 vs. 7 %; *p* < 0.05), and a decrease in biceps muscle variability where women had an increase (−23 vs. 12 %; *p* < 0.05).

**Conclusions:**

Our results suggest that women and men may not differ in the ability to perform repetitive tasks at low-to-moderate force levels. However, differences in motor control strategies employed in task performance may explain gender differences in susceptibility to developing musculoskeletal disorders when performing repetitive work for prolonged periods in occupational life.

## Introduction

Work-related MSDs have consistently been reported to occur to a larger extent among women than men (Schneider and Irastorza 2010), and especially so for the neck and shoulder/arm regions (Nordander et al. 2016). Several risk factors pertaining to biomechanical exposures at work have been associated with neck–shoulder MSDs, such as heavy lifting, forceful exertions, awkward postures, vibrations and repetitive movements (National Research Council 2001). However, the specific mechanisms for why women would be more susceptible to neck–shoulder MSDs than men, even in the same jobs, remain largely unknown. Potential reasons include differences in task allocations to men and women within the job, differences in how men and women perform the same work tasks and even differences between men and women in physiological effects when performing the same tasks in the same way (Cote 2012; Lewis and Mathiassen 2013). Most likely, all of these factors contribute in a complex interaction to explain gender differences in MSD occurrences. The focus of the present study is to understand sex-specific differences in how men and women respond to performing the same fatiguing task, as a contribution to explaining possible sex differences in biomechanical and physiological responses of relevance to MSDs. We investigate a short-cycle, repetitive manual task as a model of repetitive work occurring, for instance, in manufacturing industries, meat cutting and cashier work, which have been shown to entail an increased risk for neck/shoulder MSDs (Kilbom 1994; Cote et al. 2008; da Costa and Vieira 2010).

According to a recent review of the literature on fatigability and associated physiological mechanisms (Hunter 2014), there is still a considerable lack of understanding of the reasons for sex differences in neuromuscular function and fatigability, and the consequences of these differences to functional performance of daily tasks. Sex differences have largely been studied using controlled isometric and isotonic contractions. As reviewed in Hunter 2014, these studies have shown that women have better muscular endurance than men in isometric, sub-maximal contractions of muscles in some parts of the body such as the lower back, thighs, arms and hands but not in others such as the ankle. Sex differences have also been reported to depend on the contraction intensity; for example, women had longer time-to-exhaustion than men in contractions of the biceps muscle at 20 % of their maximal voluntary capacity (MVC), while there was no difference at 80 % MVC (Yoon et al. 2007). The type of work task has also been shown to affect sex-based fatigue responses; for instance, Clark et al. (2003) showed that women had longer time-to-exhaustion than men in a static exercise at 50 % MVC, but that women and men did not differ in the time-to-exhaustion when performing dynamic work.

However, whether these previously mentioned results are applicable, or even relevant, for understanding gender differences in performing real occupational work and the subsequent consequences on MSD risk is questionable. In realistic work involving dynamic tasks rather than isometric contractions, fatigability may be determined mainly by additional factors in motor control, such as motor coordination and the ability to vary or share effort between multiple muscles. The phenomenon of postures, movements and muscle activities varying between successive repetitions of a task (intended to be identical in performance) is referred to as ‘motor variability’ (Mathiassen 2006; Madeleine 2010; Srinivasan and Mathiassen 2012). Although motor variability has been proposed to be an important factor in determining individual differences in susceptibility to developing fatigue, pain and MSDs (Madeleine 2010; Srinivasan and Mathiassen 2012), whether systematic gender differences exist in motor variability has seldom been studied, except for a few studies (Cote 2012). For instance, Semmler et al. (1999) showed that following training, women could increase endurance time in a low-force fatiguing contraction by altering the pattern of muscle activation and using rotating/alternating motor unit activity. On the other hand, in a study on experimental shoulder pain (Falla et al. 2008), women were not able to redistribute their shoulder muscle activity as much as men, and eventually, women also reported higher perceived pain than men. A study (Fedorowich et al. 2013) reported that women with initially high motor variability in the upper trapezius muscle activity showed longer endurance times to a repetitive pointing task than those with lower variability, and that this effect of initial variability on endurance time was not observed in men, suggesting gender specificity in the relationship between variability and fatigue.

Thus, although the recent literature shows that there may, indeed, be sex differences in both structural and functional aspects of motor control, including muscle coordination and movement strategies during upper limb tasks, there are not enough studies to explain what these specific differences may be. It is also not clear how these functional aspects can explain potential gender differences in the development of short-term responses to fatigue, which may be interpreted as signs of increased risk for developing MSDs in the long term. The aims of this study were to quantify gender differences that may exist in the performance, fatigability and muscle activity responses during a fatiguing, repetitive upper-extremity task.

This study aimed to determine, for a fatiguing, repetitive upper-extremity task:

- The extent to which women and men differ in variability in the activity of selected shoulder and elbow muscles while performing the task without fatigue;
- The extent to which women and men differ in time-to-task-termination, as a measure of fatigability;
- The extent to which women and men differ in the change in amplitude and variability of activities in selected shoulder and elbow muscles (cf. 1) from the start to termination of the task.

## Methods

### Participants

This study represents analysis of data collected from recordings of the same experimental task, performed over multiple projects. The overall sample includes 113 healthy young adults (58 F and 55 M) recruited from the University student population, with average age of 27 (SD 8) years, average height of 170.3 (SD 7.5) cm and average weight of 69.1 (SD 11.8) kg. For the whole subject sample, women were on average 166 (SD 6) cm tall, and weighed 61 (SD 8.5) kg, while men were on average 175 (SD 6) cm tall and weighed 77 (SD 9) kg. The exact numbers of participants from the overall sample whose data were included for each muscle-specific analysis (cf. Sect. 2.3) were: 108 (55F and 53M) for the upper trapezius (UT) muscle, 65 (34F and 31M) for the anterior deltoid (AD) muscle, 113 (58F and 55M) for the biceps (BIC) muscle and 49 (25F and 24M) for the triceps (TRIC) muscle. These differences in sample size for different muscles were the result of an evolution of the experimental protocol over subsequent data collections. The initial protocol involved a smaller subset of muscles focusing on the biceps and trapezius; as initial findings elucidated fatigue effects that could be explained by involvement of other muscles such as the anterior deltoid and triceps, measurements of the activities of these muscles were added at a later point. Formal power calculations could not be performed prior to the start of the study as there is no current guideline or evidence in the literature of what the size of a desired detectable effect is, to determine whether there truly is a physiologically relevant gender difference in fatigue-related muscle activity responses. Post hoc power analysis (at *α* = 0.05 and *β* = 0.80) revealed that the sample sizes available in the present study led to minimum detectable differences between groups in the coefficient of variation in RMS EMG of 16 % for UT, 33 % for AD, 35 % for BIC and 70 % for TRIC, all expressed in percentage of the mean coefficient of variation among women. Participants were excluded from the study if they displayed any of the following: history of mechanical upper limb/shoulder and/or back pain or injury; history of any neurological, vestibular or other conditions affecting balance. All study participants provided written informed consent prior to participation. All participants were right-handed. Approval for the research protocol for this study was received from the Research Ethics Board of the Centre for Interdisciplinary Research in Rehabilitation (CRIR) of Greater Montreal.

### Experimental protocol

**Fig. 1:**
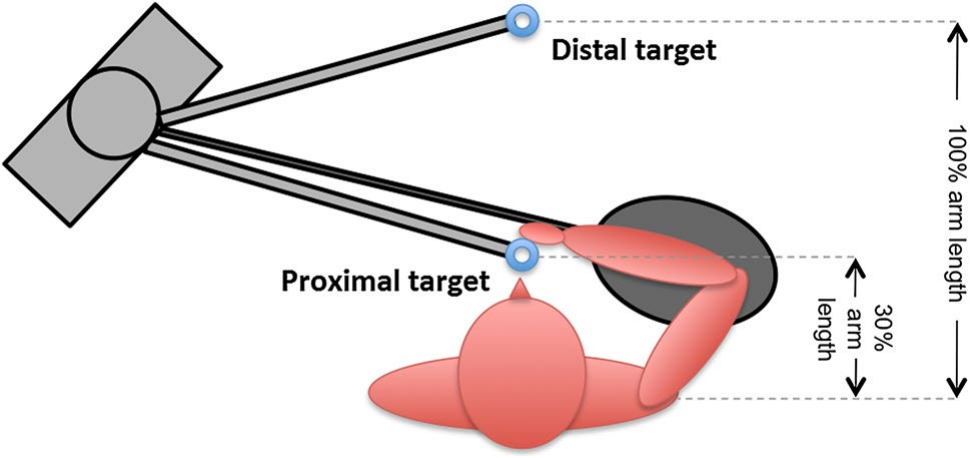
*Top view* of the experimental setup

The protocol consisted of performing a repetitive pointing task (RPT) with the aim of fatiguing the dominant arm, as first described in (Fuller et al. 2009). Briefly, participants were asked to position their feet in a standardized position, shoulder width apart. Two cylindrical touch-sensitive targets were used to guide the RPT and were adjusted to each participant’s shoulder height and aligned with their midline (Fig. 1). One target was positioned at 100 % of the participant’s arm length (distal target) and a second target was placed at 30 % of participant’s arm length (proximal target). An elliptical mesh barrier was placed under participant’s elbow joint functional range of motion to ensure that arm motion was maintained in the horizontal plane at shoulder height throughout the RPT.

The RPT required participants to perform a continuous pointing movement with the dominant arm between the distal and proximal targets while maintaining elbow position above the mesh barrier. A metronome was used to ensure the pointing task was performed at the rate of one movement per second (1 Hz); when touching the targets, the participant received auditory feedback to ensure their performance was performed at the same rate as the metronome. The RPT was performed until either the 1-Hz task frequency could not be maintained; or the participant’s elbow contacted the mesh barrier; or the first time the participant reported a perceived rate of exertion of at least 8 in the shoulder region (on the Borg CR-10 scale). Participants were not aware of the task termination criteria. Participants were asked for their rating of perceived exertion periodically, during the course of the experiment, once every minute. The first and last 30-s blocks of experimental data collected prior to protocol termination were classified as representing “No-Fatigue” (NF) and “Fatigue-Terminal” (FT) data conditions, respectively.

### Data acquisition

Upon participants’ arrival, Ag–AgCl disposable surface electrodes (Ambu™, Denmark) were placed on alcohol-cleaned and shaven skin of four muscle sites. The electrodes were applied with a center-to-center distance of 3 cm, parallel to the muscle fibers on each of the following sites: Upper Trapezius (UT: midpoint between the acromion and C7 spinous process), Anterior Deltoid (AD: vertically below the lateral end of the clavicle), Biceps Brachii (BIC: midpoint between the acromioclavicular and elbow joints), Triceps Brachii—Longhead (Midpoint and 2 cm medial of the line between posterior crista of the acromion and the olecranon) (Basmajian and Blumenstein 1980; Madeleine and Madsen 2009). EMG data were collected using a Telemyo 900 (Noraxon, USA) measurement system, and sampled at 1080 Hz.

### Analysis

#### Data analysis

All EMG data were filtered using a dual-pass fourth-order Butterworth filter (20–500 Hz). Heartbeats were removed by referencing one heartbeat per trial and cross-correlating it with the remaining signals to remove heartbeats from all muscle signals. EMG was then full-wave rectified. Root mean square (RMS) values were calculated over 30 1-s windows taken from the NF and FT conditions for each muscle signal. The RMS data were averaged over all the 30 trials to obtain one mean value for each muscle. For each of the 30 trials of the NF and FT data conditions of each participant, the cycle-to-cycle standard deviation (SD) and the cycle-to-cycle coefficient of variation (CV: standard deviation normalized by the mean value) were then computed for each muscle, and used as indices of motor variability. Time-to-task-termination was measured as the amount of time (in minutes) the task was performed by each participant.

#### Statistical analysis

Since not all the data were normally distributed, in the interest of consistency, non-parametric tests were used for all statistical comparisons. Variability during NF condition, i.e., CV of EMG amplitudes of the upper trapezius (UT), anterior deltoid (AD), biceps (BIC) and triceps (TRIC) muscles, was examined for any gender effects using Mann–Whitney *U* tests.

Non-parametric contrast values were computed according to Eq. 1 to quantify the consistency of the gender effect:

1 $$ {\text{Contrast}} = \frac{{{\text{MAD}}_{\text{bg}}^{ 2} }}{{{\text{MAD}}_{\text{bg}}^{ 2} + {\text{MAD}}_{\text{wg}}^{ 2} }} , $$

where ${\text{MAD}}_{\text{bg}}^{ 2}$ is the median squared deviation of group median values of the variable between the two gender groups, and ${\text{MAD}}_{\text{wg}}^{ 2}$ is the average of the median of squared deviations of the variable from its common median within each gender group. This contrast is a non-parametric equivalent to parametric contrasts used in previous studies (Mathiassen et al. 2005), and in the present case, it measures, on a scale from 0 to 1, the likelihood that individuals of one gender will always differ from individuals in the other gender the way that group averages predict. For instance, for variables where the median woman has a smaller value than the median man, the contrast measures the likelihood that any particular woman will have a smaller value than any particular man.

Gender effects on time-to-task-termination in the RPT were tested using a Mann–Whitney *U* test. The effects of fatigue on EMG RMS amplitude, cycle-to-cycle SD and cycle-to-cycle CV were checked by first computing the change from NF to FT conditions as a percentage of the NF value for each individual, and then using the sign test to check whether the change in each variable with fatigue was significantly different from zero. Mann–Whitney *U* tests were used to determine whether there were any gender effects in the change in EMG RMS amplitude, cycle-to-cycle SD or cycle-to-cycle CV with fatigue. *p* values less than 0.05 were considered to show statistically significant effects in all the tests.

## Results

### Gender effects on motor variability during No-Fatigue (NF) condition

The baseline variabilities in EMG amplitudes of the upper trapezius (UT), anterior deltoid (AD), biceps (BIC) and triceps (TRIC) muscles during the NF condition were examined for gender main effects using Mann–Whitney *U* tests. Table 1 shows that there were no significant gender effects on the baseline variabilities in NF condition of any of the muscle activities.

**Table 1:** CV of EMG amplitudes by gender, results of Mann–Whitney *U* tests of any gender effects in each variable and contrast values for comparison between genders during the No-Fatigue (NF) condition

Variable	Women	Men	*z*	*p*	Contrasts
	Median (inter-quartile range across subjects)	Median (inter-quartile range across subjects)			
UT CV	0.13 (0.08)	0.13 (0.07)	0.01	0.9	0.004
AD CV	0.10 (0.09)	0.09 (0.08)	1.06	0.3	0.03
BIC CV	0.17 (0.28)	0.22 (0.32)	−0.33	0.7	0.02
TRIC CV	0.13 (0.14)	0.13 (0.14)	−0.23	0.8	0.008

### Gender differences in time-to-task-termination

The task termination criterion was either a report of 8 on the Borg CR-10 scale for perceived exertion in the shoulder region, or task performance failure according to the criteria defined in the Methods section, whichever occurred first. All subjects terminated the RPT due to first reaching a perceived exertion of at least 8. The time-to-task-termination expressed as median (inter-quartile range across subjects) was 6.5 (3.75) min in women and 7 (4.0) min in men, and the difference between the two were not statistically significant (*z* = −0.35, *p* = 0.7).

### Effects of fatigue on EMG mean amplitude and variability in the overall study population

The main effects of fatigue on EMG RMS amplitude, cycle-to-cycle SD and cycle-to-cycle CV at the overall population level are described in Table 2. The table shows differences in EMG between NF and FT conditions expressed as a percentage of NF values, including results of sign tests to check whether changes were significantly different from zero. The EMG RMS amplitude increased significantly for all four muscles with fatigue, while the EMG SD increased only for UT, AD and BIC muscles and EMG CV increased only for UT and AD muscles.

**Table 2:** EMG changes with fatigue for the entire study population, expressed as the change from the NF to the FT conditions, expressed in percent of the NF value

Muscle	EMG RMS mean			EMG cycle-to-cycle SD			EMG cycle-to-cycle CV		
	Median (inter-quartile range across subjects)	*z*	*p*	Median (inter-quartile range across subjects)	*z*	*p*	Median (inter-quartile range across subjects)	*z*	*p*
UT	25.4 (60.9)	7.4	**1.1e** ^**−13**^	40.1 (98.1)	4.6	**3.8e** ^**−06**^	20.1 (62.9)	3.6	**3.0e** ^**−04**^
AD	25.7 (37.3)	5.0	**4.4e** ^**−07**^	41.7 (114.8)	3.5	**5.1e** ^**−04**^	12.2 (100.6)	2.7	**0.006**
BIC	36.9 (49.0)	7.1	**1.0e** ^**−12**^	23.9 (98.6)	2.9	**0.003**	−2.8 (70.4)	−0.6	0.56
TRIC	24.7 (39.2)	3.5	**4.6e** ^**−04**^	6.8 (105.1)	0.9	0.39	1.8 (70.6)	0.1	0.89

All statistically significant effects (*p* < 0.05) are highlighted in bold

### Gender differences in muscle activation responses to fatigue (gender vs. fatigue interaction effects)

**Fig. 2:**
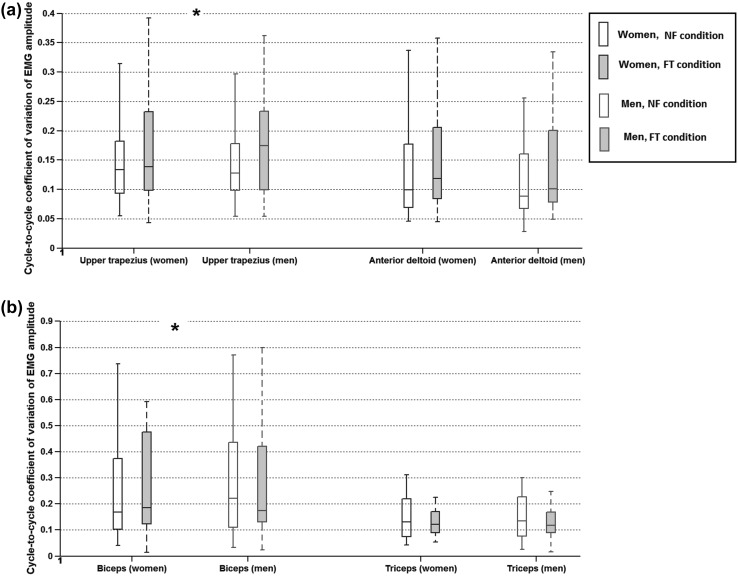
Motor variability in **a** upper trapezius and anterior deltoid muscles; **b** biceps and triceps muscles, during the No-Fatigue (NF) and Fatigue-Terminal (FT) conditions for men and women (gender indicated by color and fatigue state indicated by unfilled and filled boxes). The y-axes show the size of cycle-to-cycle coefficient of variation in amplitude of muscle activity. *Box plots* indicate median, 25th and 75th percentiles, and minimum and maximum values. *Asterisk* indicates any significant difference (*p* < 0.05) between the genders in the change from NF to FT (see* text* for more explanation)

Figure 2a, b shows the variability in EMG amplitude of UT, AD, BIC and TRIC muscles, expressed as CV, both during NF and FT conditions, stratified by gender. Gender effects on fatigue responses of EMG RMS amplitude, EMG SD and EMG CV of all four muscles are shown in Table 3, including results of the Mann–Whitney *U* test to compare within-subject CV changes between men and women. While there were no significant gender effects on change in EMG RMS amplitude with fatigue, both Table 3 and Fig. 2 show that the change in EMG variability (both SD and CV) was lower in women than in men for the UT muscle. Table 3 and Fig. 2 also show that with fatigue, while the EMG variability increased for women, it decreased for men, in the BIC muscle. Table 3 also shows the contrast values for each comparison between the two genders computed according to Eq. (1). Those significant gender effects of EMG variability with fatigue in the UT and AD muscles show moderate contrast values of around 0.3.

**Table 3:** EMG changes with fatigue by gender and muscle, expressed as the change from the NF to the FT conditions, expressed in percent of the NF value

Muscle and gender	EMG RMS mean				EMG cycle-to-cycle SD				EMG cycle-to-cycle CV			
	Median (Inter-quartile range across subjects)	*z*	*p*	Contrast	Median (Inter-quartile range across subjects)	*z*	*p*	Contrast	Median (Inter-quartile range across subjects)	*z*	*p*	Contrast
UT: Women	19.9 (56.7)	−1.4	0.2	0.09	**31.4 (72.9)**	**−2.3**	**0.02**	**0.27**	**7.2 (58.1)**	**−2.4**	**0.02**	**0.31**
UT: Men	34.9 (63.8)				**62.2 (109.4)**				**30.6 (74.3)**			
AD: Women	26.9 (42.4)	−0.4	0.7	0.01	34.6 (112.7)	−1.4	0.2	0.02	7.9 (85.1)	−1.4	0.16	0.07
AD: Men	24.7 (29.6)				46.7 (159.5)				31.2 (89.4)			
BIC: Women	34.8 (37.3)	−1.0	0.3	0.07	**51.7 (105.0)**	**3.0**	**0.003**	**0.31**	**11.8 (69.0)**	**3.4**	**6.2e** ^**−04**^	**0.33**
BIC: Men	49.1 (67.6)				**−0.5 (75.1)**				**−23.2 (63.6)**			
TRIC: Women	20.7 (34.1)	0.1	0.9	0.05	32.8 (100.3)	0.4	0.7	0.10	2.9 (65.7)	0.3	0.8	0.08
TRIC: Men	30.3 (44.5)				0.2 (114.2)				−2.1 (84.9)			

All statistically significant effects (*p* < 0.05) are highlighted in bold

## Discussion

To the best of our knowledge, this is the first study to compare motor responses accompanying fatigue between men and women in a standardized repetitive task involving the upper extremities. In contrast to earlier protocols utilized in controlled studies of gender differences in muscle activation and motor performance, this task involved muscles of both the shoulder and elbow joints at the same time and had a task performance requirement that could be satisfied using multiple coordination strategies (i.e., by utilizing the various biomechanical degrees of freedom about the shoulder and elbow joints).

In general, we found that there were no gender differences in EMG responses at baseline (Table 1), and that EMG RMS amplitude increased with fatigue in all muscles across the whole sample of individuals tested (Table 2). However, the time-to-task-termination, as defined by a rating of perceived exertion of 8 on the Borg CR-10 scale, did not differ between men and women. Similar results have been reported by earlier studies utilizing the same repetitive pointing task protocol and task termination criteria [e.g. (Fuller et al. 2009)]. However, this result is both in agreement with, and in contrast to results from the few other previous studies that have considered gender differences during fatiguing dynamic contractions. Women were less fatigable than men for a protocol of 30 maximal dynamic contractions with the knee extensor and knee flexor muscles at a relatively constant speed (Pincivero et al. 2003). Similarly, the time to task failure was longer for women than men in a dynamic task that required lifting and lowering of a load equivalent to 20 % MVC for as long as possible at the rate of 1 contraction every 3 s (Hunter 2014). However, these differences have been reported to diminish during repeated contractions at higher velocities (Senefeld et al. 2013). It is difficult to directly compare effects in men and women of tasks requiring maximal or sub-maximal isokinetic contractions or intermittent loads which are percentages of individual maximal strengths, simply because similar to the static isometric experiments, these differences may or may not exist if adjusted for strength as a covariate. Similarly, it is also difficult to directly compare results from the cited studies of endurance in sub-maximal dynamic tasks to fatigue responses in a dynamic task like ours, which was terminated prior to complete task failure. Thus, one should be careful in generalizing results from different studies as they may be highly task-specific.

Differences in performance of both static and dynamic tasks between genders have so far mainly been ascribed to: (a) differences in contractile properties associated with muscle fiber size and composition; women being reported to have a greater proportional area of type I muscle fibers than men and smaller type II fiber area in muscles such as the quadriceps, (Simoneau and Bouchard 1989), and (b) to differences in muscle perfusion and metabolism where women exhibit greater muscle perfusion than men and also show lower increase in metabolite build-up in some muscles such as the elbow flexors (Hunter and Enoka 2001). Specifically, in the trapezius muscle though, histological studies have shown that women and men have a similar fiber type composition, but women have a smaller fiber cross-sectional area than men, (Lindman et al. 1990, 1991), suggesting a lower force-generating capacity in women which may, in turn be associated with higher risks of developing neck–shoulder disorders (Meyland et al. 2014; Nordander et al. 2016).

Although the existing evidence on differences between genders in structural, morphological or physiological properties of individual muscles may, thus, offer some support to the hypothesis that women and men would differ in development of fatigue when performing a dynamic task, we did not find any gender differences in time-to-task-termination in our repetitive pointing task. This finding could be attributed to the task requiring multi-jointed movements with different possible coordination strategies: although women and men may differ in basic biological tissue properties, differences in motor control may compensate for those to the extent that there are, eventually, no differences in the ability to perform the task. For instance, women and men may utilize the available degrees of freedom and coordinate multiple muscles at the shoulder and elbow joints differently to perform the same repetitive task. The hypothesis that women and men may utilize different motor control/coordination strategies to preserve functional aspects of task performance has been previously proposed by (Cote 2012; Hunter 2014). Our findings of changes in variation of muscle activity with fatigue give some support to this hypothesis. We found that while both women and men exhibited the same variability in baseline muscle activity at the start of the task, and performed the task for similar durations, men exhibited a higher increase in trapezius muscle variability with fatigue, whereas women exhibited a greater increase in biceps muscle variability. Although only these two differences were statistically significant, the results shown in Fig. 2 and Table 3 illustrate that similar trends were also present in the anterior deltoid and triceps muscles; while men showed a greater increase in variability in the anterior deltoid activity, women showed a greater increase in variability in the triceps muscle activity with fatigue.

The results may be interpreted in consideration of the biomechanical requirements of the task. The pointing task was designed such that subjects extended their arm fully, to 100 % of their reach capacity to reach the distal target. The proximal target was positioned at 30 % of the participant’s full reach in front of them on the horizontal plane. To achieve the final reach posture, subjects were required to horizontally adduct their upper arm and extend their elbow. Consequently, the anterior deltoid and triceps muscles would be the prime movers in this task, while the trapezius muscle stabilized the shoulder and supported the weight of the arm, and the biceps would have a similar control and stabilization role for the forearm. The observed changes in variability combined with this understanding of the biomechanical demands of the task suggest that men showed a greater increase in the involvement of muscles stabilizing and moving the shoulder, while women showed a greater increase in the elbow stabilizing and prime mover muscles, thus implying a “shoulder-based” vs. an “elbow-based” control strategy in men and women, respectively, for this task.

An earlier study (Anders et al. 2004) reported that during isometric shoulder exercises (push-ups), men showed a tendency for a higher relative activation level in the shoulder muscles acting as primary movers compared to women who activated synergistic muscles that were less necessary for actual force production. The authors argued that a more goal-driven coordination pattern together with the much larger forces acting at the shoulder joint in men may lead to a higher risk for shoulder joint injuries because of a relatively less-stabilized functional configuration. In the present experimental task, since the trapezius muscle is the primary stabilizer, it may be that women implement a motor control strategy that defers the mechanical loading of the trapezius muscle down the kinetic chain (i.e., the biceps and triceps) to mitigate fatigue in the upper trapezius. These plausible alternative mechanisms (i.e., men use the shoulder more, or women use the shoulder less) highlight the need to better understand gender differences in motor control strategies: although women and men seem to have the same time-to-task-termination in this study, in prolonged performance of similar tasks in a working environment, different strategies used by women vs. men might lead to different risks of shoulder vs. elbow injuries in women and men.

Finally, while we focused on biological sex-specific mechanisms in this study of motor control and fatigue, we recognize that the occurrence and reporting of work-related MSDs in women and men will also be influenced, not only by additional factors at the individual level, such as differential reactions to pain symptoms, but also by numerous organizational, social and cultural factors. Consequently, although our study findings may provide some biological explanation for sex differences in upper-extremity occupational injuries, the work environment encountered by men and women, their interaction with that environment, and numerous factors outside work should also be considered when analyzing sex differences in upper-extremity MSDs.

## Conclusion

We did not find statistically significant sex differences in the time-to-task-termination in our fatiguing repetitive task, and this suggests that even though women and men may differ in muscle contractile properties as suggested in the literature, differences in motor control strategies may compensate to the extent that there are no sex differences in the ability to perform dynamic tasks at low-to-moderate force levels. That men seemed to use a more “shoulder-based” compensation strategy while women used a more “elbow-based” strategy, may contribute towards explaining a differential susceptibility to MSDs in women and men performing repetitive work for prolonged periods of time in occupational life.

## Abbreviations

MSD: Musculoskeletal disorders
MVC: Maximum voluntary capacity
EMG: Electromyography
SD: Standard deviation
UT: Upper trapezius
AD: Anterior deltoid
BIC: Biceps
TRIC: Triceps
RPT: Repetitive pointing task
NF: No fatigue
FT: Fatigue terminal
CV: Coefficient of variation
